# Measuring the Quality of Life in Diabetic Patients: A Scoping Review

**DOI:** 10.1155/2020/5419298

**Published:** 2020-05-20

**Authors:** Lorenzo Palamenghi, Milvia Marta Carlucci, Guendalina Graffigna

**Affiliations:** ^1^EngageMinds HUB - Consumer, Food & Health Engagement Research Center, Department of Psychology, Università Cattolica del Sacro Cuore - Milano, Italy; ^2^Faculty of Agriculture, Food and Environmental Sciences, Università Cattolica del Sacro Cuore, Piacenza, Italy; ^3^Department of Psychology, Università Cattolica del Sacro Cuore, Milan, Italy

## Abstract

**Background:**

Diabetes mellitus is a widely diffused chronic condition which impacts on several aspects of patients' lives. In the current clinical practice, the implementation in the clinical routine of monitoring systems of patients' outcomes has led to an increased generation and use of several measures for the assessment of patients' quality of life (QOL). Nevertheless, this construct appears to be particularly complex, and its operationalization is variable across different measures. The purpose of this paper is to offer an updated review of the diabetes-specific QOL measures present in scientific literature with a specific focus on the broad domains assessed.

**Methods:**

A scoping review was carried out with the purpose of identifying the existing measures in literature and describing their implicit representation of QOL in diabetes care. Five different databases (Scopus; Web of Science Core Collection; Medline; PsycInfo; and Cochrane Central Register of Controlled Trials) were searched with a string including validation studies of adult-only, diabetes-specific QOL measures. Each measure was then qualified according to its structure, a qualitative assessment of the broad domains of QOL it comprises, and finally an overview of the psychometric properties of its first validation.

**Results:**

30 scales were identified and assessed. Theme analysis shows that QOL is operationalized with multidimensional surveys comprising of both mental, physical, and social health components. Some scales also consider the impact of societal attitudes, public policies, and context on QOL.

**Conclusion:**

Several self-report measures of QOL specifically developed for diabetic patients exist in scientific literature. The present scoping review reports scales structure, broad domains of QOL, and development purpose. This may help in understanding the concept of QOL in diabetic patients and may also serve the purpose of guiding the reader in the choice of the most appropriate instrument or in the development of a new one.

## 1. Introduction

Diabetes mellitus is a widely diffused chronic condition: according to the latest edition available of the IDF Diabetes Atlas, in 2013, about 382 million people all over the world were suffering of diabetes, causing a yearly expenditure of at least 548 million US dollars [1].

Diabetes exposes people to both physical (cardiovascular diseases, neuropathy, diabetic foot, stroke, etc.) and psychological complications (e.g., depression and emotional distress); it also has a direct impact on several social aspects and, more generally, on daily life (due to, e.g., glycemic control and changes in dietary habits and in lifestyle) [2–5]. Indeed, psychosocial factors seem to be, for diabetic patients, better predictors of relevant clinical outcomes (i.e., mortality and hospitalization) than other physiological indexes generally used to assess health status such as HbA_1C_ [6, 7].

The impact of the disease and of treatment on all chronic patients' quality of life (QOL) and lifestyle is a key concern for both the patients themselves and their physicians. This is particularly relevant in the case of diabetic patients: the physical, psychological, and social burden of diabetes affects patients' self-care behaviors, disease management, therapeutic adherence, and, consequently, QOL [8].

This leads, in the current clinical practice, to a more frequent use of tools to measure the level of patients' perceived QOL. The purpose is, generally, to collect patients' inputs about their quality of life priorities and expectations, and to personalize their therapy and clinical course. In particular, the evaluation of QOL, a construct that has been defined as “a general concept that implies an evaluation of the impact of all aspects of life on general well-being” [9], seems to be of the utmost importance in order to better understand how new interventions (such as insulin pumps in the case of diabetic patients), medications, and practices affect patients' lives. The use of appropriate, up-to-date, disease-specific measures is likely to be the most suitable choice to assess the whole complexity of patients' experiences with their illness and treatment. The strive to implement in the clinical routine a systematic monitoring of patients' outcomes has led to an increased generation and use of QOL measures.

However, despite the clinical and scientific agreement about the importance of giving voice to diabetic patients about their QOL, no clear consensus exists about the exact definition of such construct, of its dimensions and, even less, of the best operationalization and best measures to use. A deeper comprehension of this construct is then necessary, in order to develop better suggestions and election criteria to orient researchers and clinicians in the selection of the most reliable and specific measurement tool according to the specific patients' population and their assessment objectives. From a psychological point of view, it is important in the assessment of QOL to consider the impact the sickness, and its treatment, on physical, social, and mental well-being, as stated by the World Health Organization [10]. However, QOL does depend not only on the presence or absence of impairments caused by a certain medical condition, [11] but also on a person's capacity to be engaged in his/her own care, a process that enables people to recover life projectuality and adjust to the medical condition [12] and that is determined not only by a person's state, but also on the environment surrounding him/her (e.g., the quality of the relationship with healthcare personnel, see [13]).

Many instruments being used for the assessment of QOL in adult diabetic patients are developed for general use with different kinds of patients and may not be suitable for assessing the specific needs and experiences of diabetic patients. A previous literature review from Speight et al. [14] categorizes “specific” and “generic” scales: “generic” measures, such as SF-12 or EQ-3D, are widespread methods to measure some aspects of QOL in different populations: they allow a reliable assessment and are the measures of election for scientific and clinical purposes when the aim is to compare different populations. Nevertheless, given their nature, they may suffer limitations in their ability to assess critical aspects related to issues specific of diabetes [15]. Specific measures, on the other hand, being developed within a framework comprising the specificities of a certain disease, are more suitable in addressing those specific aspects, burdens, and impact a certain disease has on somebody's lifestyle and QOL.

On the basis of this premise, the present literature review was carried out with the main objective of identifying the measures present in scientific literature specifically developed for the assessment of QOL in adult, diabetic patients. In particular, we were interested in the following:

1. Understanding how the construct of diabetic patients' QOL was operationalized in the development of such measurements, in order to disentangle implicit clinical and scientific representation of the phenomenon (i.e., measures content and domains of QOL assessed)
2. Furthermore, we reviewed existing QOL measures used in diabetes to propose a comprehensive and systematic descriptive framework of the methodological peculiarities of existent measures in order to support clinical choice and practice (i.e., measures structure, year and country of development, and psychometric properties of the identified measures).

Previous literature reviews of QOL measures in diabetes are more focused on either the structure and psychometric properties of the scales, possibly outdated, or reviewing measures for more specific targets (e.g., diabetes with foot problems) [14, 16–19].

## 2. Methods

For the purpose of this paper, we performed a scoping review as defined by Armstrong et al. [20]. The method of the scoping review was preferred over a more systematic approach since it allows for broader, less focused research questions. Moreover, unlike systematic reviews, often in scoping reviews inclusion criteria do not entail the quality of studies—which can be assessed post hoc: this was more in line with the main purpose of our study to better understand implicit phenomenon representation in the operationalization of the measurements tools and main gaps in diabetes-related QOL assessment. To carry out our study, we referred to the framework proposed by Arksey and O'Malley [21], a methodology which allows a transparent and rigorous, though flexible, way to collect and report evidences through a scoping review. This is a multistep process, namely

- identification of the relevant studies
- study selection
- data charting
- data report.

To better refine and circumscribe results and findings, we decided to only consider studies of first validation (hence excluding revalidations, translations, or adaptations). This choice is also related to the main focus of our analysis: i.e., disentangling the implicit representation of the QOL phenomenon in the first operationalization and conceptualization of the measurement. We also decided to only consider scales developed for adult patients. For similar reasons, and since the most recent literature review with a similar purpose we could find is dated 2009 [14], the time range considered for the research was limited from 2009 to 2019. To avoid, by the way, to only limit our research to a small sample of most recent measures, we decided to extract measures' names and references from older reviews found during the databases interrogation.

### 2.1. Identification of the Relevant Studies

To identify all possibly relevant studies, we interrogated a selection of the most important scientific databases in the medical and psychological field:

- Scopus
- Web of Science Core Collection
- Medline
- PsycInfo
- Cochrane Central Register of Controlled Trials.

Search was conducted between the 4^th^ and the 7^th^ of February 2019. The research string was composed of five different queries:

- Diabetes
- AND Quality of life
- AND Measure
- AND Development/validation
- AND NOT Exclusion criteria (to exclude translations and pediatric- or adolescent-only scales).

**Figure 1:**
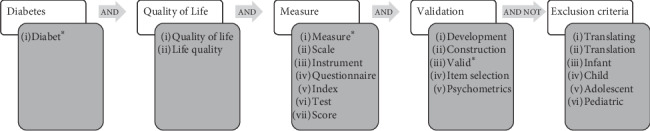
Search string. Query topics are written in light squares; synonyms used and connected by “AND” are written inside grey squares. Connectors used to connect different queries are written inside arrows.

Each query was composed of different synonyms (connected with “OR”). The exact code was different for each database, according to its peculiarities: nevertheless, the words and the logic were the same.  Figure 1 describes the queries' logic and synonyms used.

Scopus research string is shown below:

TITLE-ABS(Diabet^∗^) AND TITLE-ABS(“quality of life” OR “Life quality”) AND TITLE-ABS(Measure^∗^ OR Scale OR Instrument OR Questionnaire OR Index OR Test OR Score) AND TITLE-ABS(Development OR Construction OR Valid^∗^ OR “item selection” OR Psychometrics) AND NOT TITLE-ABS(Translating OR Translation OR Infant OR Child OR Adolescent OR Pediatric^∗^) AND (LIMIT-TO (PUBYEAR, 2019) OR LIMIT-TO (PUBYEAR, 2018) OR LIMIT-TO (PUBYEAR, 2017) OR LIMIT-TO (PUBYEAR, 2016) OR LIMIT-TO (PUBYEAR, 2015) OR LIMIT-TO (PUBYEAR, 2014) OR LIMIT-TO (PUBYEAR, 2013) OR LIMIT-TO (PUBYEAR, 2012) OR LIMIT-TO (PUBYEAR, 2011) OR LIMIT-TO (PUBYEAR, 2010) OR LIMIT-TO (PUBYEAR, 2009)).

### 2.2. Study Selection

The references downloaded from the databases were imported into a citation manager (Mendeley) which was then used for duplicate check after which two independent researchers (LP and MMC) made a title and abstract screening of the references removing papers according to the following exclusion criteria:

- Studies on nondiabetic patients
- Studies on nonadult patients
- Language of the publication different from English or unavailable online
- Publication different from first validation papers or systematic/scoping reviews.

After that, full-text selection—following the same criteria—of the studies was carried out to identify only the relevant papers. Conflicts were resolved by consensus.

### 2.3. Data Charting and Report

Finally, criteria for data extraction were defined. Scale names and data were extracted from both original and reviews. For each scale name, the first validation study was retrieved (if not already obtained during the previous steps). Validation studies were once again screened to only select those who were relevant to our research questions according to the following criteria:

- Specifically developed for diabetic patients
- Validated on an adult sample
- Validation study available in English
- Measuring quality of life or impact on quality of life: since there is not a single, acceptable definition of QOL on which there is agreement, we refrained from picking one. Moreover, since quality of life and health-related quality of life are often used interchangeably in literature [22], to avoid missing important measuring tools, we refrained from being too strict and selective towards the terminology used. We then included all such measures that explicitly make reference (in either the scale name, the study title, abstract, and authors' keywords or in the full text) to the measurement of either quality of life or health-related quality of life of diabetic patients. However, based on literature definitions of QOL [9, 23] and previous systematic reviews [14], we believe that QOL is fundamentally a multidimensional and subjective construct, comprising aspects such as mental health, physical, and/or social well-being: hence, we also decided to include some measures, identified in our literature search, that even though not explicitly developed for the assessment of QOL, they are, in our opinion, still relevant to our research since measure the impact of diabetes—or its treatment—on patients' daily life and habits. However, to avoid causing confusion in our readers, those scales will be reported separately.

We then developed the data extraction plan and database. We extracted three different types of data from validation studies:

(i) First, we carried out a qualitative theme analysis of the items comprised in the scale in order to categorize the measures according to the broad domains addressed. It is always important to have in mind what domains and aspects of QOL one wants to evaluate and to choose a measure accordingly. While most validation studies report factors and domains assessed by the scale, different authors generally use a different terminology—which could make it difficult to compare two different scales. A qualitative theme analysis of the scales' items allows to compare the broad domains assessed and the specific aspects comprised. Since no specific, systematic framework for quality of life in diabetes seems to exist, we adapted the function-neutral health-related quality of life framework developed by Krahn and colleagues [11]. This framework was developed starting from the assumption that physical functioning is not a key determinant for quality of life but that it is the relationship between the environment and the disability or illness that affects quality of life; this framework emphasizes the fact that people with a chronic condition can be healthy and have a good (or bad, indeed) quality of life—regardless of their levels of physical function; poor physical health is instead conceptualized as the presence of feelings of pain, sickness, and fatigue. This makes it a good framework for defining QOL in diabetic patients, since they generally do not experience severe physical limitations (with the possible exception of complications such as a diabetic foot). The framework identifies four core different broad dimensions plus an additional fifth (the environment), each described with several key concepts, which will be used as codes for the qualitative analysis. In brackets, details regarding the meaning of the label are specified:
  (a) Physical health:
    1. Energy/fatigue (impact of diabetes on feelings of “being fatigued,” “feeling tired” or “feeling full of energy”)
    2. Stamina (physical strength)
    3. Pain (feelings of pain)
    4. Sick/well (items regarding feeling “sick” or “ill” as opposed to feeling healthy)
    5. Rest (items assessing the quality of sleep, being capable of resting, etc.).
  (a) Mental health:
    1. Distress (feelings of mental distress)
    2. Mood (items assessing mood states, e.g., depression, happiness, and anger)
    3. Memory (and other cognitive abilities in general)
    4. Attitude (items addressing the positive/negative attitude towards the sickness or the situation)
    5. Emotional regulation (emotional response to sickness, capacity to react to diabetes-related negative events, etc.)
  (b) Social health:
    1. Social engagement (impact of diabetes on social life, e.g., going to the restaurant with friends)
    2. Relationships (impact of diabetes on existing relationship with familiars, close friends)
    3. Intimacy (impact of diabetes on sexual life)
    4. Discrimination (feeling oppressed or discriminated by others due to diabetes)
  (c) Life satisfaction/beliefs:
    1. Meaning to life (being capable of finding a meaning in one's own life regardless of diabetes)
    2. Satisfaction (towards diet, treatment, etc.)
    3. Recreation (diabetes' impact on leisure activities, hobbies, etc.)
    4. Activities (diabetes' impact on work, duties, and daily routines)
  (d) Environment:
    1. Access to services (easiness to access healthcare system, to get information, etc.)
    2. Public policies (impact of public policies on QOL, e.g., on financial situation and on out of pocket expense due to diabetes)
    3. Societal attitudes (towards diabetes).

Qualitative theme analysis was carried out by LP and MMC by inspecting scales items and searching for keywords and keywords' synonyms referring to these broad domains. Retrieved keywords were then grouped under the pertaining broad domains and reported, to allow an inspection of the domains considered within a certain measure and of their conceptualization

(ii) Then, we extracted bibliometric data, relative population, and structure of the scale; it is important while choosing a measure to be aware of the number of items (which can give a rough estimate of the time it takes to be filled), of its age and of the population it has been developed for as well as the purpose for which it was developed (clinical research, psychological screening, outcome measure of treatments, etc.)
(iii) Finally, psychometric properties were assessed according to the guidelines provided by Terwee and colleagues [24].

## 3. Results

**Figure 2:**
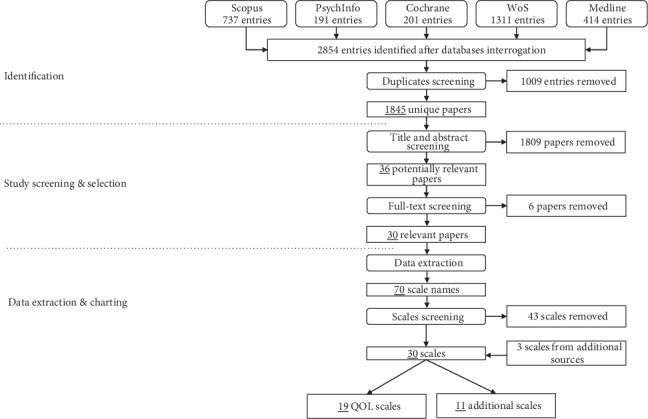
Identification and screening process. From the starting 1845 unique papers, 30 relevant papers were selected after abstract and full-text screening. From those papers, 70 scale names were extracted, of which 27 were selected for analysis. Three additional scales from other sources were also added to our study.

A synthesis of the identification and screening process can be found in Figure 2. We report our study using the Preferred Reporting Items for Systematic reviews and Meta-Analyses extension for Scoping Reviews (PRISMA-ScR) [25].

### 3.1. Identification of the Relevant Studies and Eligibility

The interrogation of the databases returned 2854 papers, which were later reduced to 1845 papers after checking for duplicates.

The screening of the titles and abstracts of the identified 1845 papers led to the selection of 36 papers potentially relevant for the purpose of the current study. Further screening of the full-text led to the subsequent removal of other 6 papers, leading to a total of 30 included papers eligible for data extraction. From these 30 papers, 70 scale names (with the corresponding references) were extracted. Each scale was briefly screened, and 27 scales were selected—according to the aforementioned criteria—for a more in-depth analysis. Three additional scales were also included in our results, derived from additional sources retrieved by the authors.

### 3.2. Data Charting and Report

#### 3.2.1. Qualitative Categorization

Tables 1 and 2 summarize the scale structure, bibliometrics, and qualitative labels for QOL measures and additional measures, respectively, while Tables 3 and 4 show the number of occurrences of each label.

Every screened measure addresses at least two different broad domains, four on average. In particular, amongst the QOL measures, each one of them comprises at least an item referring to either the life satisfaction/beliefs broad domain, mental health, or social health with very few exceptions (the W-BQ28 not comprising social health and both DDRQOL-R-9 and DMQOL not including mental health items), while less of the included measures offer an assessment of physical health (13 out of 19). Regarding the impact of the environment or of the context on diabetic patients' QOL, most measures comprise some items regarding either the financial burden, access to services, or societal attitude, the only exceptions being DQOL, DTR-QOL, and W-BQ28. One label appears to be particularly relevant for the assessment of QOL in diabetes, which is “activities,” present in 16 out of 19; these labels represent all the items that are intended to measure a disruption in daily activities and the burden of the illness (or of the therapy, at times) on work on other daily routines. Also, items regarding distress or the impact of diabetes on relationships are common.

For what concerns the “additional” measures we included in our study, assessing the impact of diabetes on daily life, there are indeed some differences. While some domains such as mental health are well assessed by all measures (with a particular focus on distress and mood) and some labels such as “activities” are very frequent as well, most of these measures do not seem to capture the importance of the environment, with only 3 measures (DCP, DDS, and PAID) assessing either the easiness of access to services, the financial burden, or societal attitude. Nevertheless, for what concerns the 4 core broad domains, there are no big differences, even though the domain of social health is possibly underrepresented, when confronted with QOL measures, with only 6 measures investigating some aspects of social life and, in particular, diabetes' impact on relationships (DCP, DDS, DIMS, DMRSQ, PAID, QSD-R, and TRIM-D).

#### 3.2.2. Bibliometrics and Structure

From the selected scales, 3 are specifically validated for Type 1 patients only and 5 for Type 2 patients only; the remaining are validated on mixed samples or the specificity is not declared.

The oldest scale retrieved is from 1988 (Diabetes Quality of Life [31]), while the most recent are from 2017 [28, 35, 42] and 2019 [29]. On average, identified measures have 37 items with a high variability, ranging between the 7-items Appraisal of Diabetes Scale [43] and the 234-items Diabetes Care Profile [44]. Three scales (DHP-1, JAPID-QOL, and ViDa1 [40, 42, 46]) have been specifically developed for Type 1 patients, while six (AsianDQOL, DMRSQ, MDQ, PAM-D, W-BQ28, and PRO-DM-Thai [26, 38, 39, 41, 49, 50]) have been developed for Type 2 patients only; all the other measurements have no specific population target.

#### 3.2.3. Psychometrics

Since no golden standard exists for the measurement of QOL, criterion validity was not assessed. Minimal Important Change (MIC) was not reported for any of the examined evaluation studies; for this reason, it has been impossible to report responsiveness. However, floor and ceiling effects were at times reported, which can provide some insight into the ability of the measurement tool to report change. While for the (arguably) most fundamental characteristics—internal consistency, construct, and content validity—most of the studies were well reported and met the criteria; the same cannot be said about test-retest reliability, floor/ceiling effects, and, in particular, interpretability.  Table 5 reports in detail the psychometric properties of each analyzed measure.

## 4. Discussion

Many measures specific for the assessment of QOL in diabetic patients or assessing the disruption of diabetes on daily life exist in scientific literature, suggesting that—in order to avoid an unnecessary development of new instruments—it would be more suitable to choose an already existing and validated measure. The present review reports the broad domains of QOL assessed by diabetes-specific measures in order to help understanding how this complex, multifaceted construct is being measured by validated, scientific tools. Each instrument included in this review is a self-report scale, mostly consisting in various items grouped into different domains or subscales. This is coherent with the definition of QOL, a multidimensional and subjective construct [9, 14, 23], described as “an individual's perception of their position in life in the context of the culture and value systems in which they live and in relation to their goals, expectations, standards and concerns” [55]. The qualitative theme analysis (summarized in Tables 3 and 4) shows that all the broad domains of QOL are well covered from existing instruments, even though only 12 measures seem to address all the 5 domains at least to a certain degree. Considering only QOL measures, physical health seems to be the broad domain assessed by the smaller number of measures: this may actually say something interesting about the domains on which diabetes has a stronger impact and is probably due to the peculiarities of diabetes, which often requires a change in dietary and lifestyle routines or an adaptation of the daily schedule due to therapy (i.e., insulin shots) which, in turn, could be disruptive towards someone's relationships, social life, etc. Those items regarding the disruption of social routines and the interference with family and friends (labeled as “social engagement,” “relationships,” and “intimacy,” surveying the impact of diabetes on the capacity of enjoying social life, on relationships with close one, and on sexual life, respectively) were indeed frequent. Items regarding distress were also found very often during the scales screening. The items assessing some aspects of the environment around the patient generally focus on societal attitude towards diabetes, while only few of them address aspects such as the financial burden of the illness (which is something that directly depends on healthcare public policies) or access to and support from healthcare services and professionals.

While in QOL measures the role played by Environment seems to be well represented, it is the most underrepresented domain amongst the additional measures we considered: this is probably the main difference between these two sets of measures, representing the importance of the context in which a patient lives for his/her QOL, while being of less importance when considering the impact of diabetes or treatment on his/her distress levels or disruption of daily life activities.

Indeed, even though it is not considered a core domain of QOL by the function-neutral framework, but an ancillary domain [11], it is still of particular importance for a comprehensive assessment of the impact of diabetes and—more generally—chronic conditions, on patients' QOL; how supportive and easy to access the healthcare system is, the policies which can ease or exacerbate the financial burden and the general societal attitude towards the illness are all potential barriers or facilitators of patients' QOL: failing to take into consideration the environment and the society in which a person lives may mean that we are missing some relevant and fundamental pieces of information on how he/she actually perceives the quality of his/her life. This is particularly evident today, within the increasing debate about patient engagement promotion and the in the light of the shared clinical consensus about the importance of increasing patients' ability to participate in their care journey and to maintain an active role in their reference community [56].

Moreover, it is worth noting that while aspects regarding mental health are as much considered as those regarding physical health, the psychological representation of illness—namely its cognitive, attitudinal, emotional, and symbolic value—the patients' sense of hope or positiveness and his/her capacity or intention to attribute a meaning to the illness and to participate actively to his/her own care planning are rarely investigated and taken into consideration. This may imply that—at least to a certain extent—the underlying rationale behind QOL measurement is to assess the amount or the absence of negative aspects related to QOL, while ignoring the amount or presence of positive aspects and behaviors (such as patients' willingness to engage in active participation). This appears poorly in line with the increasing complexity of QOL self-assessment according to patients' experience: QOL cannot be reduced to the elimination of disease symptoms and therapy side effects, but it relies also on the level of psychological elaboration and acceptance of the disease condition mastered by the patient [12]. Measuring patients' hope and positive attitude towards the disease, thus, should be a priority in diabetes QOL evaluation.

Most of the analyzed validation studies are well reported and describe measures that have good psychometric properties, even though some relevant and important information are often missing (i.e., floor/ceiling effect, interpretability, and test-retest reliability). By the way, it is worth noting that almost every validation study described the process of item generation as starting from, or integrating patients' points of view (collected through either focus groups, interviews etc.); this good practice of patient participation to measures development is important since it contributes to content validity and, in particular, in the case of QOL, since it is a construct that—by definition, as already remarked—is subjective.

Another important aspect to consider when evaluating the feasibility of using a certain measure in one's own study or clinical practice is the setting of validation: scales developed too many years ago—or in different cultures—may not actually be representative of today's life with diabetes, patients' needs, and perspectives. Also, it is important to keep in mind the context and the purpose for which the specific tool was developed: was it intended for clinical use, or instead for research? Is it patient-centered, treatment-centered, or diet-centered? While a clinically oriented measure could be used for research and vice-versa, different purposes generally require different characteristics.

One final remark worth discussing regards measures' length: while quality of life is certainly a complex construct, composed of several components that may require a consistent number of items for a thorough assessment, it is also necessary to keep in mind the burden that a very long questionnaire (such as the 200+ items DCP [44]) exerts on the patients. While a perfect balance between length and completeness may be impossible to obtain, it is necessary to keep in mind the domains that are most important for one's own purpose while selecting the most appropriate instrument, in order to avoid the application of unnecessarily cumbersome measures. This aspect risk is a potential hindrance to the systematic adoption of QOL measurements in real-word healthcare systems. Particularly in the light of PROMS and PREMS debate, the issue of making patients' assessment and input collection a continuous, rigorous and systematic practice is a forthcoming goal. However, the risk to create a “questionnaire fatigue” due to the length and complexity of QOL scales and related construct is clinically and ethically problematic. The assessment of QOL in diabetes patients should be a strategic asset to improve patients' care and cannot be transformed in an extra burden for patients, their clinicians, and the healthcare organization itself.

Finally, Table 6 provides an overview of the included measures which may be helpful in selecting the most suitable measure for one's own purpose. In particular, in order to guide the choice, we divided both QOL and additional measures according to their length and whether or not they are comprehensive of all the broad domains of QOL. Shorter measures and, in particular, those who cover all domains are generally more suitable for clinical screening and whenever there is a concern for patients' burden: nevertheless, it should be noted that while a short, comprehensive measure is indeed interesting since it allows to assess all domains with a relatively little burden, the use of such measures may also imply that those domains are being assessed only superficially or partially. Longer measures or more specific measures instead could be more suitable for those researchers who are interested in more in-depth and extensive assessment of some aspects of QOL. Additional scales could also prove useful, in particular when there is an interest in assessing the impact of diabetes or treatment on some specific aspects of someone's life as distress, or daily life.

## 5. Limitations

This scoping review has some limitations: first, we searched only those databased which we believed to be the most relevant to our research questions; second, even though we included precedent literature reviews in order to compensate for this, we decided to limit our research to the last 10 years, in order to obtain a manageable set of data. Additionally, our search was limited to such studies that have been published in English and for adult patients. This means that, potentially, relevant validation studies may have been missed by our review process. The use of an exclusion criteria (AND NOT) in the search string may have led to a bias in the identification of relevant studies.

By the way, the main purpose of our research was to identify how the QOL construct has been operationalized in the context of diabetic care. We chose to analyze only the evidences from original, first validation studies, excluding further evidences of psychometric robustness: this probably means that for at least some of the described measures, some evidences regarding psychometric properties have been missed. The validation of a measurement tool is an iterative and complex process that generally requires several evidences and studies: considering only the first evidences of validity is limiting, and our analysis should not be considered comprehensive of this aspect, as this is not the principal aim of this paper. The assessment of psychometric evaluation was carried out according to the guidelines from Terwee et al. [24] which, even though robust and systematic, have been updated by those provided by the COSMIN initiative [57].

## 6. Conclusions

Many self-report measures of QOL specifically developed for diabetic patients exist in scientific literature, suggesting that before an effort is done to develop a new measure, an attempt should be done to select an already existing and validated instrument, in order to avoid useless duplication and redundancy. This scoping review reports broad domains of QOL that are assessed by measuring tools available in scientific literature specifically developed for adult diabetic patients. All the four core broad domains of QOL seem to be covered by existing measures. Nevertheless, there seems to be a lack of measures including an assessment of the environment around the patient and/or of his positiveness/positive behaviors. This also suggests the need for further validation studies aimed at developing more complete assessment instruments. The medium length of the scale retrieved, however, also suggests the need for future attempt to reduce the length of used measures and or to develop shorted and more user-friendly ones.

Finally, for the purpose of guiding the reader in the choice of the most suitable measure, other information such as scales structure, development purpose, and some first evidences of psychometric validation were reported.

If an adequate measure could not be found in literature such that it suits the needs of a certain specific purpose, the results from the qualitative theme analysis could be used in order to understand which aspects of QOL are most commonly assessed in diabetic patients and which, on the other hand, are currently superseded in order to guide the development of a new measure.

## Figures and Tables

**Table 1:** QOL scales structures and broad domains.

Name	Scale					Broad domains				
	First publintdcation year	No. of items	Subscales/domains	Population (*n*, language, diabetes type)	Development purpose	Physical health	Mental health	Social health	Life satisfaction/beliefs	Environment
Asian diabetes quality of life questionnaire (AsianDQOL) [26]	2015	21	Financial; diet; memory and cognition; energy; relationship	136 (English version), English-Chinese-Malay, Type 2	QOL assessment in multiethnic/multilanguage Asian populations	Energy/fatigue	Memory, mood	Relationships, intimacy	Activities	Policies (financial burden)
Audit of diabetes-dependent quality of life (ADDQOL-19) [27]	1999	19	Employment/career; social life; family relationships; friendships; sex life; sport/leisure; travel; future (own); future (family); motivation; do physically; others fuss	152, English	Patient-centered assessment of diabetes impact on QOL (both research and clinical practice)	Energy/fatigue	Distress	Relationships, social engagement, intimacy	Recreation, activities	Societal attitudes
Diabetes diet-related quality of life revised (DDRQOL-R) [28]	2017	17	Satisfaction with diet; burden of diet therapy; perceived merits of diet therapy	184 (115 test-retest), Japanese	Assessment of the effect of dietary therapy on daily lifestyle and QOL		Attitude (towards diet)	Social engagement (diet-related)	Satisfaction (diet-related)	Policies (financial burden)
Diabetes diet-related quality of life revised—short form (DDRQOL-R-9) [28]	2017	9	Satisfaction with diet; burden of diet therapy; perceived merits of diet therapy	Same sample as DDRQOL-R	Short version of DDRQOL-R more feasible for clinical purposes			Social engagement (diet-related)	Satisfaction (diet-related)	Policies (financial burden)
DAWN2 Impact of Diabetes Profile (DIDP) [29]	2019	7		2207, Australian both Type 1 and Type 2	Assessment of perceived impact of diabetes on QOL	Sick/well	Emotional regulation	Relationships	Recreation, activities, satisfaction (diet)	Policies (financial burden)
Diabetes Obstacles Questionnaire (DOQ-30) [30]	2016	30	Relationship with medical professionals; support from friends and family; knowledge of the disease; lifestyle changes; exercising; self-monitoring;uncertainty about a consultation; medication; insulin-use	853, cross-country (Estonia, France, Belgium, Serbia, Slovenia, Turkey)	Assessment of diabetes-related QOL addressing a variety of obstacles		Distress	Relationships	Activities	Access to services (healthcare), societal attitude
Diabetes quality of life (DQOL) [31]	1988	46	Satisfaction; impact; worry: diabetes related; worry; social/vocational	136 adults, 56 adolescents, English	QOL assessment, originally for controlled trials	Sick/well, rest, pain	Distress, mood	Relationships, intimacy	Satisfaction, activities, recreation	Societal attitude
Diabetes quality of life (DQOL) [32]	2012	16	Emotional suffering; social functioning; adherence to the treatment regimen; diabetes-specificsymptoms	402, Korean	QOL assessment	Sick/well	Mood, distress	Relationships	Recreation, activities	
Diabetes quality of life brief clinical inventory (DQOL-Brief) [33]	2004	15		498, English	Treatment-focused QOL assessment in clinical practice	Pain, sick/well, rest	Distress	Relationships, intimacy	Satisfaction, activities	Societal attitude
Diabetes Quality of Life Clinical Trials Questionnaire, Revised (DQLCTQ-R) [34]	1999	57	Physical function; energy/fatigue; health issues; mental health; DQOL satisfaction; DQOL impact; DQOL social worry; DQOL diabetes worry; worry (Hypoglycemia Fear Survey); treatment satisfaction; treatment flexibility; social stigma; frequency of symptoms; bothersomeness of symptoms	942 cross-country (Canada, France, Germany, US)	QOL assessment in multinational clinical trials	Sick/well, stamina, energy/fatigue, pain	Distress, mood, emotional regulation	Social engagement, relationships, intimacy, discrimination	Activities, satisfaction, recreation	Societal attitude
Diabetes-specific quality of life questionnaire module (DMQOL) [35]	2017	10		170, Chinese (available also in English)	Diabetes-specific module to be used along with WHOQOL-BREF			Relationships, social engagement	Activities, satisfaction, recreation	Policies (financial burden), societal attitude
Diabetes therapy-related quality of life (DTR-QOL) [36]	2012	29	Burden on social activities and daily activities; anxiety and dissatisfaction with treatment; hypoglycemia; satisfaction with treatment	284, Japanese	Assessment of influence of treatment on patients' QOL	Pain, sick/well	Distress	Relationships, social engagement	Activities, satisfaction (general and with treatment)	
Diabetes-39 (D-39) [37]	1997	39	Energy and mobility; diabetes control; anxiety and worry; social burden; sexual functioning	427, English	QOL assessment	Energy/fatigue, sick/well	Distress	Relationships, intimacy	Activities	Societal attitude
Multidimensional Diabetes Questionnaire (MDQ) [38]	1997	41	Interference; severity; support; positive reinforcing behaviors; misguided support behaviors; self-efficacy; outcome expectancies	249, French, Type 2	Understanding individual differences in adjustment to diabetes		Distress	Relationships, social engagement	Activities, recreation	Societal attitude, access to service (healthcare)
The 28-item well-being questionnaire (W-BQ28) [39]	2012	28	Generic negative well-being; energy; generic positive well-being; generic stress; diabetes-specific negative well-being; diabetes-specificstress; diabetes-specific positive well-being	353, English, Type 2	QOL assessment in clinical and research practice	Energy/fatigue, rest	Emotional regulation, mood, distress		Meaning to life, activities, satisfaction	
The Japanese insulin-dependent diabetic patient quality of life scale (JAPID-QOL) [40]	2011	19	Diabetes status-related; support of family or community-related; social acceptance-related	105, Japanese, Type 1	QOL assessment in clinical trials with beta cell replacement therapy	Sick/well, rest	Distress	Relationships	Satisfaction (with treatment), activities	Societal attitude
The patient-reported outcomes instrument for Thai patients with Type 2 diabetes mellitus (PRO-DM-Thai) [41]	2015	44	Physical function; symptoms; psychological well-being; self-care management; social well-being; global judgments of health; satisfaction with care and flexibility of treatment	500 (study 1), 200 (study 2), Thai, Type 2	Tool for healthcare providers to investigate treatment outcomes	Sick/well	Distress, mood	Relationships, social engagement	Satisfaction, activities	Access to services (healthcare, public transport)
The ViDa questionnaire for Type 1 diabetes (ViDa1) [42]	2017	34	Interference in life; self-care; well-being; worry about the disease	578, Spanish, Type 1	QOL assessment in both research and clinical practice	Rest, sick/well, pain	Distress, emotional regulation	Discrimination, intimacy, social engagement	Activities, satisfaction (with treatment), recreation	Societal attitude

**Table 2:** Additional scales (impact of diabetes or treatment on daily life) structures and broad domains.

Name	Scale					Broad domains				
	First publication year	No. of items	Subscales/domains	Population (*n*, language, diabetes type)	Development purpose	Physical health	Mental health	Social health	Life satisfaction/beliefs	Environment
Appraisal of Diabetes Scale (ADS) [43]	1991	7		200, English	Assessment of diabetes-related distress		Distress, mood		Meaning to life	
Diabetes Care Profile (DCP) [44]	1996	234	Control problems; social and personal factors; positive attitude; negative attitude; self-care ability; importance of care; self-care adherence; diet adherence; medical barriers; exercise barriers; monitoring barriers; understanding mgt. practice; long-term care benefits; support attitudes	352, English	Assessment of factors important in a patient's adjustment to diabetes and its treatment in daily life	Energy/fatigue	Attitude, mood	Relationships	Activities, recreation	Policies (financial burden), access to services (healthcare)
Diabetes Distress Scale (DDS) [45]	2005	17	Emotional burden; physician-related distress; regimen-related distress	167 (Honolulu); 137 (US); English	Measure of diabetes-related emotional distress for use in research and clinical practice	Energy/fatigue	Distress, mood, emotional regulation	Relationships	Satisfaction	Access to services
Diabetes health profile (DHP-1) [46]	1996	32	Psychological distress; barriers to activity; disinhibited eating	2239, English, Type 1	Identify psychosocial dysfunction of patients in an ambulatory care setting	Pain	Emotional regulation, mood, distress		Activities, recreation	
Diabetes health profile—18 (DHP-18) [47]	2000	18	Psychological distress; barriers to activity; disinhibited eating	426 English, 460 Danish	Adaptation of DHP-1 for Type 2 patients		Emotional regulation, mood		Activities, recreation	
Diabetes Impact Measurement Scale (DIMS) [48]	1992	44	Diabetes-specific symptoms; nonspecific symptoms; combined symptoms; well-being; diabetes-related morale; social role fulfillment	130 (52 test-retest), English	Measurement of health status	Stamina, energy/fatigue, sick/well, rest, pain	Distress, mood	Intimacy, social engagement, relationships	Activities, meaning to life	
Diabetes Medication System Rating Questionnaire (DMRSQ) [49]	2012	54	Convenience satisfaction; negative events; interference; self-monitoring of blood glucose burden; efficacy; social burden; positive mood; negative mood; well-being; treatment satisfaction; treatment preference	537 (482 test-retest), English, Type 2	Assessment of treatment burden/experience on patients	Energy/fatigue, sick/well, pain (using treatment)	Distress (treatment-related and general), mood, emotional regulation	Intimacy, relationships	Satisfaction (with treatment), activities	
Perceptions about medications for diabetes (PAM-D) [50]	2009	37	Scheduling flexibility; portability convenience; regimen inconvenience; medication effectiveness; difficulty remembering medications; gastrointestinal side effects; hypoglycemia-related side effects; weight/edema side effects; emotional side effects	343, English, Type 2	Understanding multidimensional perceptions about patients' treatment preferences and burden	Rest, pain, sick/well	Emotional regulation, distress, mood, memory		Activities	
Problem Areas in Diabetes scale (PAID) [51]	1995	20		451, English	Assessment of adjustment to diabetes	Energy/fatigue	Distress, mood	Relationships		Societal attitude
Questionnaire on stress in patients with diabetes-revised (QSD-R) [52]	1997	45	Leisure time; depression/fear of future; hypoglycemia; treatment regimen/diet; physical complaints; work; partner; doctor-patient relationship	1930	Assessment of stress in patients	Sick/well	Distress, mood	Relationships	Recreation, activities	
Treatment-Related Impact Measure Diabetes (TRIM-D) [53]	2009	55	Treatment burden; daily life; diabetes management; compliance; psychological health; device function; device bother	507, English	Assessment of the impact of treatment and delivery systems/devices on patients' functioning, well-being and QOL		Emotional regulation, distress, mood	Social engagement, relationships	Activities, satisfaction (with treatment)	
Treatment-Related Impact Measure—Nonsevere Hypoglycemic Events (TRIM-HYPO) [54]	2015	33	Daily functioning; emotional well-being; diabetes management; sleep; work productivity	407, English	Assessment of the impact of hypoglycemic events on daily life	Stamina, rest	Memory, emotional regulation		Activities, recreation	

**Table 3:** Summary of broad domains and labels of QOL measures.

Broad domains	No. of measures including the domain	Labels	No. of measures including the label
Physical health	13	Energy/fatigue	5
		Pain	5
		Stamina	1
		Rest	5
		Sick/well	10

Mental health	17	Distress	13
		Mood	6
		Memory	1
		Attitude	1
		Emotional regulation	4

Social health	18	Relationships	14
		Intimacy	7
		Social engagement	9
		Discrimination	2

Life satisfaction	19	Meaning to life	1
		Activities	16
		Recreation	8
		Satisfaction	12

Environment	16	Policies	5
		Societal attitude	10
		Access to services	3

**Table 4:** Summary of broad domains and labels of additional measures.

Broad domains	No. of measures including the domain	Labels	No. of measures including the label
Physical health	8	Energy/fatigue	5
		Pain	4
		Stamina	2
		Rest	3
		Sick/well	4

Mental health	11	Distress	9
		Mood	11
		Memory	2
		Attitude	1
		Emotional regulation	7

Social health	6	Relationships	7
		Intimacy	2
		Social engagement	2
		Discrimination	0

Life satisfaction	11	Meaning to life	2
		Activities	9
		Recreation	5
		Satisfaction	3

Environment	3	Policies	1
		Societal attitude	1
		Access to services	2

**Table 5:** Validation studies psychometrics. The sign “+” (plus) means the scale meets the requirements; “−” (minus) means it does not; “?” (interrogation mark) means it meets criteria only partially or that methodology is not clear; and “0” (zero) means no information were found.

Scale name	Content validity	Internal consistency	Construct validity	Test-retest reliability	Interpretability	Floor/ceiling
Appraisal of Diabetes Scale (ADS) [43]	?	+	+	?	0	0
Asian Diabetes Quality of Life Questionnaire (Asian DQOL) English version [26]	+	+	+	?	0	0
Audit of diabetes-dependent quality of life (ADDQOL-19) [27]	+	+	+	0	0	0
Diabetes Care Profile (DCP) [44]	?	−	+	0	0	0
Diabetes diet-related quality of life revised (DDRQOL-R) [28]	+	+	+	−	0	0
Diabetes diet-related quality of life revised—short form (DDRQOL-R-9) [28]	+	+	+	−	0	0
Diabetes Distress Scale (DDS) [45]	+	+	+	0	0	0
Diabetes health profile (DHP-1) [46]	+	+	+	0	0	+
Diabetes health profile-18 (DHP-18) [47]	+	+	+	0	0	+
Diabetes Impact Measurement Scale (DIMS) [48]	—	—	+	?	0	0
DAWN2 Impact of Diabetes Profile (DIDP) [29]	+	+	+	0	0	+
Diabetes Medication System Rating Questionnaire (DMRSQ) [49]	+	+	+	+	0	−
Diabetes Obstacles Questionnaire (DOQ-30) [30]	+	+	+	0	0	0
Diabetes quality of life (DQOL) [31]	+	?	+	?	0	0
Diabetes quality of life (DQOL) [32]	+	+	+	0	0	0
Diabetes quality of life brief clinical inventory (DQOL-Brief) [33]	+	?	?	0	0	0
Diabetes Quality of Life Clinical Trials Questionnaire, Revised (DQLCTQ-R) [34]	+	—	+	—	0	0
Diabetes-specific quality of life questionnaire module (DMQOL) [35]	+	+	+	0	0	+
Diabetes therapy-related quality of life (DTR-QOL) [36]	?	+	+	+	0	+
Diabetes-39 (D-39) [37]	+	+	+	0	0	0
Multidimensional Diabetes Questionnaire (MDQ) [38]	+	+	?	0	0	0
Perceptions about medications for diabetes (PAM-D) [50]	+	+	+	+	0	−
Problem Areas in Diabetes Scale (PAID) [51]	+	?	+	0	0	0
Questionnaire on stress in patients with diabetes-revised (QSD-R) [52]	+	+	+	?	0	0
The 28-item well-being questionnaire (W-BQ28) [39]	?	+	+	0	+	−
The Japanese insulin-dependent diabetic patient quality of life scale (JAPID-QOL) [40]	+	?	+	0	0	+
The patient-reported outcomes instrument for Thai patients with Type 2 diabetes mellitus (PRO-DM-Thai) [41]	+	+	+	0	0	0
The ViDa questionnaire for Type 1 diabetes (ViDa1) [42]	+	+	+	?	0	0
Treatment-Related Impact Measure Diabetes (TRIM-D) [53]	+	+	+	+	+	+
Treatment-Related Impact Measure—Nonsevere Hypoglycemic Events (TRIM-HYPO) [54]	+	+	+	+	0	+

**Table 6:** Measures' overview.

		QOL measures	Additional measures
Short (≤20 items)	Comprising all five domains	ADDQOL-19; DIDP; DQOL-Brief; JAPID-QOL	DDS
	NOT comprising all five domains	DDRQOL-R; DDRQOL-R-9; DQOL; DMQOL	ADS; DHP-18; PAID

Long (>20 items)	Comprising all five domains	AsianDQOL (Type 2); DQOL; DQLCTQ-R; D-39; PRO-DM-Thai; ViDa1	DCP
	NOT comprising all five domains	DOQ-30; DTR-QOL; MDQ (Type 2); W-BQ28 (Type 2)	DHP-1 (Type 1); DIMS; DMRSQ (Type 2); PAM-D (Type 2); QSD-R; TRIM-D; TRIM-HYPO

## Data Availability

Data are available upon request to the corresponding author.
